# The disappearance of white matter in an adult-onset disease: a case report

**DOI:** 10.1186/s12888-020-02551-x

**Published:** 2020-03-27

**Authors:** Cyrus SH Ho, Simone Mangelsdorf, Mark Walterfang

**Affiliations:** 1 Department of Psychological Medicine, National University Health System, Level 9, NUHS Tower Block, 1E Kent Ridge Road, Singapore, 119228 Singapore; 2 Neuropsychiatry Unit, Royal Melbourne Hospital, Melbourne, Australia; 3 Melbourne Neuropsychiatry Centre, University of Melbourne and North-Western Mental Health, Melbourne, Australia; 4 Florey Institute of Neuroscience and Mental Health, University of Melbourne, Melbourne, Australia

**Keywords:** Vanishing white matter disease, Adult-onset, Psychiatric, Cognitive, Neurological

## Abstract

**Background:**

Vanishing white matter disease (VWMD) is one of the most prevalent hereditary white matter diseases in childhood, but it is increasingly recognised in adulthood with high phenotypic variation and severity.

**Case presentation:**

We report a case of an adult female presenting with emotional lability and cognitive impairment, in addition to progressive dystonia, ataxia, postural instability and recurrent falls. Magnetic resonance imaging (MRI) of the brain and genetic testing confirmed the diagnosis of VWMD.

**Conclusions:**

VWMD has a broad clinical presentation in adulthood, and the age at onset of symptoms is one of its most important prognostic factors. It is crucial to recognize the pathognomonic MRI patterns and consider VWMD as a differential diagnosis when assessing patients presenting with psychiatric, cognitive and non-specific neurological symptoms.

## Background

Vanishing white matter disease (VWMD) is a progressive leukodystrophy involving the white matter of the brain exclusively. It is caused by autosomal recessive mutations in one of the five eukaryotic translation initiation factor 2B (EIF2B) genes involved in protein translation initiation and protein synthesis regulation, of which EIF2B5 mutations are the most common [1]. This results in the derangement of the cellular-stress-response, particularly disrupting myelination and affecting both astrocytes and oligodendrocytes while sparing the neurons [2], and rapid, severe neurological deterioration is often provoked by stressful situations such as infection, minor head trauma, acute psychological stress or sudden fright [3, 4].

Although its incidence is unknown, VWMD—also known as childhood ataxia with central hypomyelination—is believed to be one of the most common hereditary diseases affecting the white matter in childhood [2]. It clinically ranges from antenatal, infantile, early childhood to juvenile-onset form, and the disease course is variable [5]. The classical and most common form that occurs in childhood at age 2 to 6 years of age usually presents with significant chronic progressive cerebellar ataxia, spasticity that is less notable, epilepsy and comparatively mild intellectual decline [6]. Nevertheless, prominent systemic involvement resulting in serious complications of encephalopathy, hepatosplenomegaly, pancreatitis, renal dysplasia, cataracts and shunted growth can occur in the early onset and severe variants of this disease [7]. In general, the prognosis is grave, with the majority of patients dying after a few years. However, some die only after several months, and some manage to survive for several decades [6].

However, VWMD is increasingly recognized in adulthood, manifesting large phenotypical and severity variability, with the latest known onset of disease to be 55 years [8]. Late-onset VWMD has been found to correspond to 15% of all described cases [9]. Adult-onset symptoms include complicated migraines, seizures, spasticity, cerebellar ataxia [6], psychiatric manifestations of mood disturbances and psychosis [10] and dementia [11]. Females may suffer from premature ovarian insufficiency regardless of the severity of the disorder, although an association with primary ovarian dysfunction, identified as ovarioleukodystrophy, is a rare occurrence [12]. With the wide range of presentation seen in late-onset VWMD, it can be challenging to diagnose clinically.

Here, we highlight a case of an adult patient presenting to the psychiatric clinic with emotional lability and cognitive impairment, in addition to motor incoordination, and who was eventually shown to have VWMD. The patient’s brother provided informed consent to the publication of this report.

## Case presentation

In July 2010, a 43-year-old woman presented to our clinic with a 1-year history of vestibular symptoms, progressive ataxia, postural instability and recurrent falls, which started after sustaining a shock when falling on the kerb while preparing a cigarette. She noted then that her “stomach muscles just did not do what they were supposed to” to maintain her posture. Her medical history included a diagnosis of anxiety–depression a decade prior, but she was no longer on medication. She did not have any family history of neurological or psychiatric disorder. She reported a depressed mood, increasing irritability, personality change with uncontrollable anger and disinhibition with coprolalia, and cognitive decline. Her uncle noted significant short-term and working memory problems, a reduced span of attention and a reduced capacity for the higher-level organization of her affairs, mainly financial matters.

Neurological examination revealed a dystonic gait, with dystonia more noticeable in the lower than upper limbs. She also had diffuse hyperreflexia, dysmetria, and bilateral non-habituating palmomental and pollicomental reflexes. On neurocognitive examination, she scored 27.5/30 on the Mini-Mental State Examination and 89.5/100 on the Neuropsychiatry Unit Cognitive Assessment Tool (NUCOG). Of note, her scores on most domains other than language (attention, memory, executive function and spatial function) were about 0.5 standard deviation below the mean—lower than what would be expected given her educational background of higher school certification. Notably, she was unable to recall three items, but recalled all with a categorical prompt, suggesting a more frontal-executive origin to her memory difficulties. The formal neuropsychological assessment indicated marked impairments in processing speed, working memory, delayed recall and executive function. Her memory difficulties were secondarily affected by her executive dysfunction, whereby she had retrieval difficulties which were aided by prompting. Her blood investigations, including full blood count, renal panel, liver function test and thyroid function test were normal. Her hormonal assays were unremarkable. Magnetic resonance imaging (MRI) of her brain revealed diffuse white matter abnormality (Panel A) with rarefaction and cystic degeneration replaced by fluid (Panel B), and a radiating stripe-like pattern indicative of residual tissue strands (Panel C) involving anterior and posterior areas. However, the grey matter was preserved. Genetic testing was done, and she tested positive for a homozygous mutation in the EIF2B5 gene. Given the classical neuroimaging and genetic findings, she was diagnosed with vanishing white matter disease (VWMD).

With no definitive treatment, the patient deteriorated physically, necessitating nursing home care 3 years after the initial onset of symptoms. According to her uncle whom we contacted in late 2018, she continued to remain in the nursing home, but her condition was stable.

## Discussion and conclusions

This case illustrated the apparent wide phenotypic range in VWMD that was discussed earlier. The limited number and small longitudinal studies on the natural history of VWMD have nevertheless revealed that the age at onset is one of the most relevant prognostic determinants [11]. Those with a presentation at less than 4 years of age have a significant age at onset impact on the disease trajectory, typically characterized by a rapidly progressing path dominated by motor dysfunction. In contrast, those with a presentation from 4 years onwards is correlated with a disease progression independent of age at onset, and is consistent with a more stable and diverse path with reduced mortality [13]. However, there is a considerable amount of variability within these two categories, with an exceptionally slow progressive illness experienced in some patients with early-onset of symptoms, and some suffering rapid deterioration and demise in those with the late-onset disease [13]. This is in contrast to other leukodystrophies in which the age of onset of symptoms is inversely correlated with clinical severity, with the disease course more protracted in those with a later onset [14]. Furthermore, seizures and episodic worsening are significant determinants of disease progression for all ages of onset [6], emphasizing the prominence of proper seizure management and precautionary measures such as antipyretics, vaccinations, preventing head injuries and maintaining emotional stability for all patients.

Our patient’s late onset of symptoms at the age of 42 in the absence of seizures and stress-provoked episodes (other than her initial episode that led to her symptom onset) likely accounted for her favourable prognosis and slow disease progression. Initial presenting symptoms were neurological in most patients, as compared to psychiatric and cognitive symptoms manifesting in 11–16% of adult-onset VWMD and up to 24% of all patients, with neurological symptoms typically occurring within the next few years [5, 9]. As illustrated in our patient, adult-onset VWMD exhibits earlier and more prominent cognitive problems as compared to early-onset disease with predominantly more motor disability, and this is consistent with other leukodystrophies [15].

**Fig. 1:**
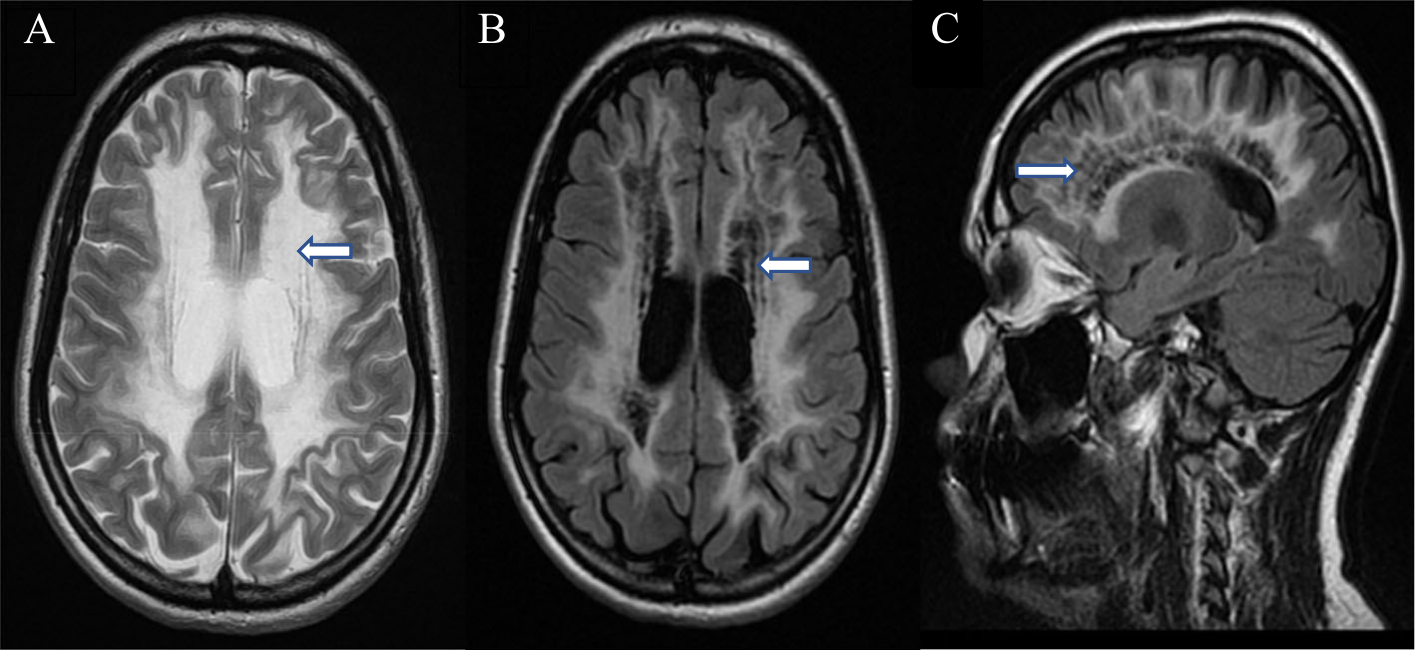
T1 and FLAIR images of the patient with vanishing white matter disease. **a** Axial T1-weighted image showing the diffuse abnormality of the white matter. **b** Axial FLAIR image showing the rarefaction and cystic degeneration of the white matter. **c** Sagittal FLAIR image showing the radiating stripe-like pattern within the white matter, indicative of residual tissue strands

Diagnosis of VWMD is made primarily with MRI due to the pathognomonic imaging patterns, which are highly sensitive and specific for the disease (refer to Fig. 1) [6]. This can be especially helpful when there is limited access to genetic testing and a lack of other reliable biomarkers which can be challenging to obtain (glycine [16] and asialotransferrin [17] obtained only via the cerebrospinal fluid). In cases of chronic progressive neurological deterioration, with MRI showing rarefaction and cystic degeneration within diffuse white matter abnormalities, it is pertinent to differentiate VMWD from other causes of leukodystrophy, particularly mitochondrial leukoencephalopathies. VWMD has a more widespread white matter aberrancy with increased diffusivity on diffusion-weighted imaging and no contrast enhancement. However, mitochondrial leukoencephalopathies tend to contain regions of restricted diffusion and exhibit focal contrast enhancement [18].

While VWMD is a rare neurodegenerative disease, it is not difficult to diagnose due to the pathognomonic MRI patterns, which are highly sensitive and specific for the disease, provided that physicians are aware of the existence of this disease. As psychiatrists and other physicians may be the first point of contact for individuals with neuropsychiatric symptoms, it is important that a comprehensive neurological and neuroradiological evaluation is carried out. VWMD or other leukodystrophies should be considered as part of a differential diagnosis for a patient with mood disturbances, cognitive impairment and motor disorders, for which the identification of white matter rarefaction on MRI would lead to a diagnosis of VWMD. Through early detection of this condition, it is possible to facilitate preventive measures to reduce episodes of rapid decline induced by stress, which can be critical in retarding the disease progression.

## Data Availability

All data generated or analyzed during this study are included in this published article.

## Abbreviations

VWMD: Vanishing white matter disease
EIF2B: Eukaryotic translation initiation factor 2B

## References

[1] Leegwater PA, Vermeulen G, Könst AA, Naidu S, Mulders J, Visser A (2001). Subunits of the translation initiation factor eIF2B are mutant in leukoencephalopathy with vanishing white matter. Nat Genet.

[2] van der Knaap MS, Breiter SN, Naidu S, Hart AA, Valk J (1999). Defining and categorizing leukoencephalopathies of unknown origin: MR imaging approach. Radiology.

[3] van der Knaap MS, Barth PG, Gabreëls FJ, Franzoni E, Begeer JH, Stroink H (1997). A new leukoencephalopathy with vanishing white matter. Neurology..

[4] Vermeulen G, Seidl R, Mercimek-Mahmutoglu S, Rotteveel JJ, Scheper GC, van der Knaap MS (2005). Fright is a provoking factor in vanishing white matter disease. Ann Neurol.

[5] Accogli A, Brais B, Tampieri D, La Piana R (2019). Long-standing psychiatric features as the only clinical presentation of vanishing white matter disease. J Neuropsychiatry Clin Neurosci.

[6] van der Knaap MS, Pronk JC, Scheper GC (2006). Vanishing white matter disease. Lancet Neurol.

[7] van der Knaap MS, van Berkel CGM, Herms J, van Coster R, Baethmann M, Naidu S (2003). eIF2B-related disorders: antenatal onset and involvement of multiple organs. Am J Hum Genet.

[8] Gascon-Bayarri J, Campdelacreu J, Sánchez-Castañeda C, Martínez-Yélamos S, Moragas M, Scheper GC (2009). Leukoencephalopathy with vanishing white matter presenting with presenile dementia. J Neurol Neurosurg Psychiatry.

[9] Labauge P, Horzinski L, Ayrignac X, Blanc P, Vukusic S, Rodriguez D (2009). Natural history of adult-onset eIF2B-related disorders: a multi-centric survey of 16 cases. Brain J Neurol.

[10] van der Knaap MS, Leegwater PA, van Berkel CGM, Brenner C, Storey E, Di Rocco M (2004). Arg113His mutation in eIF2Bepsilon as cause of leukoencephalopathy in adults. Neurology.

[11] Prass K, Brück W, Schröder NW, Bender A, Prass M, Wolf T (2001). Adult-onset Leukoencephalopathy with vanishing white matter presenting with dementia. Ann Neurol.

[12] Fogli A, Rodriguez D, Eymard-Pierre E, Bouhour F, Labauge P, Meaney BF (2003). Ovarian failure related to eukaryotic initiation factor 2B mutations. Am J Hum Genet.

[13] Hamilton EMC, van der Lei HDW, Vermeulen G, Gerver JAM, Lourenço CM, Naidu S (2018). Natural history of vanishing white matter. Ann Neurol.

[14] Van Haren K, Bonkowsky JL, Bernard G, Murphy JL, Pizzino A, Helman G (2015). Consensus statement on preventive and symptomatic care of leukodystrophy patients. Mol Genet Metab.

[15] Vanderver A (2016). Genetic Leukoencephalopathies in adults. Contin Minneap Minn.

[16] van der Knaap MS, Wevers RA, Kure S, Gabreëls FJ, Verhoeven NM, van Raaij-Selten B (1999). Increased cerebrospinal fluid glycine: a biochemical marker for a leukoencephalopathy with vanishing white matter. J Child Neurol.

[17] Vanderver A, Schiffmann R, Timmons M, Kellersberger KA, Fabris D, Hoffman EP (2005). Decreased asialotransferrin in cerebrospinal fluid of patients with childhood-onset ataxia and central nervous system hypomyelination/vanishing white matter disease. Clin Chem.

[18] Finsterer J, Zarrouk MS (2012). Leukoencephalopathies in mitochondrial disorders: clinical and MRI findings. J Neuroimaging Off J Am Soc Neuroimaging.

